# Unconventional Secretion and Intercellular Transfer of Mutant Huntingtin

**DOI:** 10.3390/cells7060059

**Published:** 2018-06-14

**Authors:** Bor Luen Tang

**Affiliations:** 1 Department of Biochemistry, Yong Loo Lin School of Medicine, 117597 Singapore, Singapore; bchtbl@nus.edu.sg; Tel.: +65-6516-1040; 2 NUS Graduate School for Integrative Sciences and Engineering, 117456 Singapore, Singapore

**Keywords:** Huntingtin (HTT), Huntington’s disease (HD), membrane traffic, polyglutamine (polyQ) tract, tunneling nanotube (TNT), unconventional secretion

## Abstract

The mechanism of intercellular transmission of pathological agents in neurodegenerative diseases has received much recent attention. Huntington’s disease (HD) is caused by a monogenic mutation in the gene encoding Huntingtin (HTT). Mutant HTT (mHTT) harbors a CAG repeat extension which encodes an abnormally long polyglutamine (polyQ) repeat at HTT’s N-terminus. Neuronal pathology in HD is largely due to the toxic gain-of-function by mHTT and its proteolytic products, which forms both nuclear and cytoplasmic aggregates that perturb nuclear gene transcription, RNA splicing and transport as well cellular membrane dynamics. The neuropathological effects of mHTT have been conventionally thought to be cell-autonomous in nature. Recent findings have, however, indicated that mHTT could be secreted by neurons, or transmitted from one neuronal cell to another via different modes of unconventional secretion, as well as via tunneling nanotubes (TNTs). These modes of transmission allow the intercellular spread of mHTT and its aggregates, thus plausibly promoting neuropathology within proximal neuronal populations and between neurons that are connected within neural circuits. Here, the various possible modes for mHTT’s neuronal cell exit and intercellular transmission are discussed.

## 1. Introduction

Age-associated neurodegenerative diseases that affect central nervous system (CNS) neurons are typically sporadic with only a small fraction traceable to inherited mutations in susceptibility genes. Huntington’s disease (HD), on the other hand, is a prototypical hereditary monogenic disorder. The disease is autosomal dominant, typically midlife onset and is presented with movement disorders, cognitive decline and psychiatric symptoms with progressively fatality [1,2]. Symptoms of the disease becomes obvious typically in the 30s–40s, and these symptoms gradually worsen over the course of one or two decades before the eventual demise of the patient. Typical HD symptoms include involuntary, jerky motions, which are usually preceded by a general lack of movement coordination and unsteady gait in the earlier years. HD patients also exhibit psychiatric symptoms, progressive cognitive decline and eventually full-blown dementia. HD is a member of a larger group of trinucleotide repeat expansion diseases [3], and its underlying mutation is an expansion of the trinucleotide CAG repeat within exon 1 of the Huntingtin (*HTT*) gene, which translates into an abnormally long N-terminal glutamine (polyQ) tract of the mutant HTT (mHTT) protein. Healthy subjects harbor stretches with 6–35 glutamine residues in length, but this could be extended up to hundreds of residues in HD individuals. The length of mHTT’s polyQ tracts correlate well with the age of disease onset and the severity of symptoms in patients. Individuals with >40 CAG repeats would usually be considered to harbor a full-penetrance disease allele, and disease symptoms are invariably in place for these individuals.

The HD susceptibility gene *HTT* is a vital gene for vertebrate embryonic development, and its targeted deletion in mice results in early embryonic lethality with gastrulation defects [4,5,6]. *HTT* encodes a large 348 kDa polypeptide contains multiple HEAT repeats (named after its presence in four proteins, Huntingtin, Elongation factor 3, α subunit of protein phosphatase 2A and Target of rapamycin 1) [7], which forms alpha solenoid structures capable of forming molecular scaffolds [8,9,10]. Accordingly, HTT has many known cellular binding partners and implicated roles in a large number of cellular processes [11]. HTT’s interactions with proteins such as Huntingtin-associated protein 1 (HAP1) [12,13] and Huntingtin-interacting protein 1 (HIP1) [14] regulate endocytic transport, while its engagement of motor proteins of the kinesin family and dynein [15,16] facilitates microtubule-based cytoplasmic, axonal and dendritic transport. HTT is also localized to the nucleus, where it interacts with multiple transcription factors in transcriptional complexes [17,18,19]. HTT is known to have a role in processes such as mitotic spindle orientation [20] (Godin and Humbert, 2011), biogenesis of the primary cilium [21,22,23] and autophagy [10,24]. All the above could be linked to HTT’s function in neurogenesis and nervous system development [20,22,25,26]. It is therefore possible that the disruption of normal HTT function by the extended polyQ stretch could have a pathogenic role in HD neurons. 

Neuropathology in HD has, however, been principally attributed to the polyQ repeat extension found in the N-terminus of mHTT, as transgenic mice expressing only the 5′ fragment containing an expanded repeat recapitulated HD features [27]. Such fragments could be generated in two ways. The extended CAG repeat could cause aberrant splicing of exon 1 of *HTT*, resulting in a short polyadenylated mRNA that is translated into an exon 1-encoded polypeptide [28]. HTT is also enriched in proline (P), glutamic acid (E), serine (S), and threonine (T) (PEST) sequences and is thus susceptible to cleavage by a number of disease-related or pathologically upregulated proteases, including calpain, caspases and lysosomal proteases [29,30,31,32,33,34,35,36]. The polyQ-bearing N-terminal fragments generated are prone to oligomerization and the formation of fibrillary aggregates [37], which may be resistant to clearance [38]. Transcriptional inhibition when the polyQ tract containing mHTT fragments is translocated into and aggregates in the nucleus is thus a major pathological mechanism in HD. Perturbations of cytoplasmic and axonal transport processes as well as nucleocytoplasmic transport [39,40] also contribute to neuronal pathology. Although aggregation-prone polyQ-containing mHTT N-terminal fragments could recapitulate the disease in experimental models, the relative roles of full-length and fragmented mHTT in the human disease are, however, still not fully understood.

The primary neurological symptoms of HD result from the disruption of neural circuits between the cerebral cortex and the striatum—the corticostriatal pathway. Neurons in the striatum, particularly the medium spiny projection neurons, are the earliest and most affected by HD pathology. Several possibilities have been put forth to explain this specific susceptibility. Firstly, striatal neurons are highly dependent on brain-derived neurotrophic factor (BDNF) from cortical projections, and BDNF expression [41], as well as its axonal transport and delivery to the cortico-striatal synapses, is dependent on HTT [42]. Furthermore, endocytosis, axonal retrograde transport and signaling from the BDNF-bound tropomyosin receptor kinase B (TRKB) receptor-bearing signaling endosome in neurons are also dependent on HTT [43]. Secondly, a striatum-enriched small GTP-binding protein, Rhes [44], binds mutant HTT and promotes sumoylation of the latter, thus increasing its toxicity [45,46]. The combined pathological contributions of transcriptional and protein transport disruption by mHTT could therefore be the primary causes underlying striatal neurons’ particular vulnerability to mHTT neurotoxicity.

With the above in mind, mHTT pathology has been conventionally thought to be neuronal cell autonomous, although striatal astrocytes have also been shown to contribute to neuronal HD pathology [47]. However, accumulating evidence suggests that many toxic oligomers or aggregates that are associated with different neurodegenerative diseases could be secreted extracellularly and transferred intercellularly via extracellular microvesicles [48,49] and tunneling nanotubes (TNTs) [50,51]. Recent findings have also shown that cytoplasmic mHTT or its aggregates could exit neurons, or be transmitted from one neuronal cell to another [52,53,54,55] via different modes of unconventional secretion [56] and TNT [57]. Furthermore, secreted or intercellularly translocated mHTT could initiate or seed mHTT aggregation in recipient neurons, and has been postulated to drive disease progression in a prion-like fashion [55,58,59]. In the paragraphs below, I shall discuss what is currently known about how cytoplasmic mHTT could exit a neuronal cell, or be otherwise transmitted to other neurons or glia. 

## 2. Unconventional Secretion and Intercellular Transport of Neuropathogenic Factors

Most of the common neurodegenerative diseases have unique and well-recognized pathogenic proteins derived from over-expressed or mutant forms of key proteins, or their proteolytic products. In general, these are prone to misfolding, olgomerization and the formation of amyloid or fibrillary aggregates, which could overwhelm the capacities of the ubiquitin–proteasome pathway (UPS) [60] or the autophagy machinery [61], thus impairing clearance. Interestingly, these pathological proteins and their oligomers/aggregates can be found extracellularly, independent of neuronal death and cell lysis. Accumulating evidence over the past decade has suggested that these extracellular secreted pathological entities could propagate disease when taken up by neighboring neurons and glia. As these proteins largely exist cytoplasmically upon translation and lack recognizable leader or signal sequences for targeting to the classical secretory pathway [62,63], their mode of secretion is necessarily unconventional [64,65,66], occurring largely via the generation of extracellular vesicles (EVs) such as exosomes [67,68]. On the other hand, the transfer of such proteins between cells could also occur via a more direct route—through intercellular tubular connections, termed TNTs [50,69,70].

EVs are a heterogeneous group of membrane-bound vesicles with sizes ranging from 10 nm to a few µm in diameter [67]. The best studied amongst these in terms of the nervous system and neurodegenerative diseases, are the exosomes and ectosomes [71,72]. Exosomes are 50–100 nm vesicles first generated via intraluminal budding into multivesicular bodies (MVBs) and subsequently released when MVBs fuse with the plasma membrane [67,68]. Ectosomes are larger particles (typically >100 nm, up to 500 nm) that are shed directly from the plasma membrane sites [73] such as the cilia [74]. Even larger membrane-bound extracellular structures such as apoptotic bodies (>500 nm) and oncosomes (1–10 µm) could be generated from dying cells and tumors [75], respectively. The EVs are known to carry a range of cargo, from small molecules to DNA/RNA to polypeptides, and are associated with a myriad of physiological as well as pathological transmission roles [48,49]. The TNTs, first described by Gerdes’ laboratory [69], are a heterogeneous bunch of actin-based tubular extensions that form physical connections between cells. The larger and more sophisticated amongst these are open-ended conduits and could serve to transfer macromolecules and organelles such as mitochondria [76,77] and lysosomes [78,79]. 

Perhaps the most extensively documented extracellular secretion of pathological molecules are those associated with the most prevailing age-associated neurodegenerative diseases, namely Parkinson’s disease (PD) and Alzheimer’s disease (AD). α-Synuclein [80], the major component of Lewy bodies found in PD and other α-synucleinopathies [81] is secreted extracellularly. The unconventional secretion of α-Synuclein occurs via extracellular vesicles [82,83], with exosomes most likely being the main vehicle [84,85]. Such secretion is enhanced by mutation [86] and promoted by neuronal activity [84,87,88] as well as autophagy failure or inhibition [89,90,91] and stress signaling [92,93]. Secreted α-Synuclein could be taken up via endocytosis by neighboring cells [85,94,95] and could presumably transmit neurotoxicity in this manner [90,96]. Intercellular transfer of α-Synuclein via TNTs has also been documented [79,97]. Exosomes could mediate the extracellular release of amyloid beta peptides formed in endosomes [98]. Tau [99], the hyperphosphorylated form of which is a component of the neurofibrillary tangles found in AD and other tauopathies, is also unconventionally secreted via EVs and exosomes [100,101]. In this case secretion is enhanced in certain proteolytic products [102] and by neuronal activity [103,104]. Again, the intercellular transfer of tau via TNTs has been observed [50,105]. Pertaining to AD, another protein that is known to be unconventionally secreted is insulin degrading enzyme (IDE) [106,107], which other than insulin also degrades the amyloid β-peptide (Aβ). IDE secretion via unconventional secretion has been shown to underlie the effect of statins in reducing the amyloid load [108,109]. Another neurodegenerative disease with known secreted pathological molecules is amyotrophic lateral sclerosis (ALS). Both wild-type and ALS mutant forms of the Cu-Zn superoxide dismutase (or SOD1) can be secreted in an unconventional manner [110,111,112,113]. This is likewise the case for two other ALS disease proteins, the RNA-binding protein transactive response DNA binding protein of 43 kDa (TDP-43) [114] and fused in sarcoma (FUS) [115], which is secreted via exosomes, with the former also transmitted between neurons across axon terminals [116] as well as via TNTs [117]. 

Finally, it should be noted that unconventional secretion via exosomes [118] and other EVs, as well as intercellular transfer via TNTs [119], has been documented earlier with the prion protein Scrapie (PrP^Sc^), the pathological agent for transmissible spongiform encephalopathies. Abnormally folded PrPSc could multiply in cells by interacting with and driving the conformational conversion of cellular PrP^C^ to PrP^Sc^. The cellular mechanisms responsible for the cell-to-cell spreading of prions has recently been reviewed in detail by Vilette and colleagues [120].

A summary of the major neurotoxic agents that are unconventionally secreted or otherwise intercellularly transferred is provided in Table 1.

## 3. Externalization and Intercellular Transfer of mHTT

HTT is ubiquitously expressed in human tissues and mHTT can be found in plasma and cerebrospinal fluid (CSF) [122]. Cytoplasmic mHTT can be translocated extracellularly, and multiple lines of evidence suggest that it can be transferred from cell to cell and inter-neuronally within the CNS. Fibrillar polyQ peptide aggregates in the media could be internalized by mammalian cells in culture, upon which these could gain access to the cytosolic compartment and become co-sequestered in aggresomes [121]. In a human 293T cell culture model over-expressing HTT-exon 1 polyQ-Green fluorescent protein (GFP) fusion constructs, both the CAG-repeat containing RNA and the polyQ-GFP is found incorporated into EVs, and the latter could be taken up by a mouse striatal cell line [123]. In a *Drosophila* model where a human mutant N-terminal *HTT* fragment with a 138 residue polyQ tract was specifically expressed in olfactory receptor neurons (ORNs), polyQ-containing protein aggregates accumulate at the synaptic terminals, and these progressively spread throughout the fly brain. These aggregates are internalized via endocytosis and accumulated within the neurons of neighboring, as well as remote, brain regions, and caused the demise of some of the more susceptible neurons [53]. Not all polyQ-bearing aggregates spread well in this model, as a polyQ-expanded HTT exon 1 fragment and a truncated ataxin-3 with a pathogenic polyQ expansion formed aggregates at the ORN terminals but did not really go beyond this. Interestingly, genetic analyses also indicated that the extracellular release and spread of aggregates requires two membrane trafficking components, namely N-ethylmalemide–sensitive fusion protein 1 (NSF1), a general factor required for soluble NSF attachment protein receptor (SNARE)-mediated intracellular membrane traffic, autophagy and synaptic vesicle fusion, as well as dynamin, which is required for endocytosis. Furthermore, the transfer of mHTT between host and grafted brain tissues appear possible, as mHTT aggregates were found postmortem in intracerebral fetal neural allografts made previously in several HD patients [124]. The above findings attested to the transfer of mHTT aggregates to neighboring cells, especially those connected by a neuronal circuit, but also to more remote locations from the source.

In an extensive study using the R6/2 HD mouse model [27], the transneuronal propagation of mHTT was investigated in several experimental settings, including human embryonic stem cell (hESCs)-derived neurons integrated within organotypic brain slices of the R6/2 HD mouse, an ex vivo mixed-genotype cortico-striatal slice culture and transmission within the HD mouse corticostriatal pathway in vivo [52]. In the first setting, hESCs were differentiated into NPCs, marked by GFP and microinjected with R6/2 HD mouse brain slices. These appeared to form functional synaptic connections with neurons from the brain slices. mHTT from R6/2 brain tissues spread to the cytoplasm and nuclei of human neurons and resulted in pathological manifestations in terms of morphological changes to cell soma and neurites. Interestingly, this mouse–human interneuronal transfer of mHTT is inhibited by botulinum toxins, which cleaves the synaptic SNAREs [125], thus suggesting the involvement of synaptic vesicle release-like mechanisms. Similar observations were also made with human neurons stereotactically injected into a R6/2 mouse brain. In mixed-genotype corticostriatal brain slice co-cultures, where tissues from the R6/2 cortex and wild-type mouse striatum were combined, the formation of an extensive network of corticostriatal connections could be observed (but such connections failed to form in a mixed culture of R6/2 striatum and wild-type cortex). mHTT could spread from the R6/2 cortex to wild-type striatal medium spiny neurons (DARPP-32-positive MSNs) that were remotely located from the corticostriatal contact area. mHTT that had spread in vivo within the corticostriatal pathway of mice was investigated by co-injection with viral vectors driving the expression of Q72-Htt-Exon1 and synaptophysin-GFP into the cortex of wild-type mice. In this way, the presynaptic termini of exogenous mHTT expressing cortical neurons connected to the striatum could be marked. Areas with and without a synaptophysin-GFP–labeled network of presynaptic terminals could be discerned in the striatum. Areas with high levels of synaptophysin-GFP labelling contained a high proportion of DARPP-32-positive MSNs displaying intracellular mHTT aggregates, whereas those with low synaptophysin-GFP signals had correspondingly low number of MSNs with mHTT aggregates. mHTT expressed in cortical neurons could therefore spread via its striatal projections to striatal neurons. In another study, the injection of HD patient-derived fibroblasts or induced pluripotent stem cells (iPSCs) generated from these resulted in progressive gliosis and inflammation, striatal neuron loss as well as HD-like motor and cognitive impairment in wild-type mice [55]. These studies affirmed that transfer of mHTT between tissues is possible and that it could likely be a mode of disease propagation from affected to healthy neurons. 

Interneuronal transfer of mHTT could also occur via TNTs [57]. Aggregates that formed from mHTT with 68Q repeats, formed after expression by transient transfection, could be efficiently transferred between differentially-labeled CAD neural cell lines independent of release by dying cells. This transfer required or was enhanced by cell–cell contact, and fluorescently-labelled aggregates could be visualized in TNTs that form between cells. Interestingly, the TNT connection between cells could be moderately enhanced by mHTT-68Q over that of a lower repeat number (mHTT-17Q). TNT-mediated mHTT-68Q transfer also occurred with cultured primary cerebellar granule neurons and aggregates, and TNT-like connections were apparently observed between neurons and contaminating astrocytes. These findings indicate that mHTT, like α-Synuclein and tau, could spread intercellularly, between neurons and between neurons and glia, via direct physical connections. 

## 4. Mechanisms Underlying the Extracellular Secretion of mHTT—Exosomal, Autophagic or Lysosomal Secretion?

How exactly does the unconventional secretion of mHTT peptides or their aggregates occur? From the findings described above, the exact mechanism of secretion has remained unclear. Although the involvement of exosomes is always suspected, whether exosomes are a major mode of extracellular secretion of mHTT remains unclear. There is evidence for mHTT secretion in exosomes. In a study of the transmission of mHTT from HD patient fibroblasts, mHTT is found within exosome marker-containing microvesicles that are release by these fibroblasts, and these could be taken up by cultured cells [55]. In another report, tissue transglutaminase 2 was shown to promote the assembly of a protein complex that included mHTT and the exosomal markers ALG-2-interacting protein X (ALIX), tumor susceptibility gene 101 protein (TSG101), as well as the BCL2-associated athanogene 3 (BAG3+, a co-chaperone involved in the clearance) of mHTT. The assembly of this complex may facilitate the selective recruitment of mHTT into exosomes [126]. On the other hand, mHTT and its aggregates have also been shown to be absent in exosomes [127]. The requirement for membrane fusion factors NSF [53] and SNAREs [52] demonstrated in some reports could imply a few different mechanisms. One possibility would be release by a SNARE-requiring mechanism akin to synaptic vesicle exocytosis. However, no clearly-defined mechanism that is able to accommodate large mHTT aggregates is currently known. 

Another prominent unconventional secretion mechanism that has emerged recently involves autophagy [128] and the Golgi reassembly stacking protein (GRASP) [63,64,65]. This pathway is responsible for the unconventional secretion of both membrane [129] and cytosolic proteins [130,131,132]. The exact mechanism underlying this pathway is yet unclear, but it could involve the fusion of amphisomes generated from autophagosome with the plasma membrane [133]. However, although mHTT aggregates do induce autophagy [134], there is no evidence that mHTT secretion is dependent on autophagy. On the other hand, a very recent report on the unconventional secretion of cytoplasmic fatty acid binding protein 4 (FABP4) has illustrated the existence of another pathway that is independent of GRASP and autophagy, but involves cargo enclosure within endosomes and secretory lysosomes [135]. Regulated exocytosis based on lysosome-like organelles has been documented extensively in immune cells [136], but even conventional lysosomes in many cell types could apparently fuse with the plasma membrane in response to increases in intracellular free Ca^2+^ concentration in a calcium sensor synaptotagmin-dependent manner (reminiscent of synaptic vesicles in axon terminals) [137]. 

In this regard, the importance of an unconventional lysosome secretion pathway for mHTT was indeed demonstrated recently [56]. Trajkovic and colleagues found that mHTT expressed in Neuro2A cells is secreted preferentially over wild-type HTT in a manner that is both brefeldin-A and Arf1 dominant-negative mutant insensitive (the latter factors disrupt the endoplasmic reticulum–Golgi transport required for classical secretion). Interestingly, extracellular mHTT in this case was found largely in a soluble form (i.e., remaining in the supernatant after a 100,000× *g* centrifugation). Inside the cell, a portion of the mHTT was found within membranous compartments, the bulk of which colocalized with the late endosome/lysosome (LE/Lys) markers, Lysosome-associated membrane protein (Lamp) 1 and 2. The silencing of synaptotagmin 7 reduced mHTT secretion from Neuro2A cells as well as a striatal cell line and primary cortical neurons, as did chemical cross-linking-based ablation of the LE/Lys compartments. The authors found that mHTT is preferentially was targeted to LE/Lys compared to wild-type HTT, although this lysosomal exocytosis is not likely a major mHTT clearance pathway. mHTT secretion is sensitive to inhibitors of neutral sphingomyelinase (NS) and phosphatidylinositol 3-kinase (PI3K), both of which appeared to be important for the endosomal targeting of mHTT. Therefore, other than the TNTs, lysosomal-based secretion is the most-documented mode of mHTT unconventional secretion.

## 5. Prion-Like Spread of mHTT Aggregates?

Prion protein (PrP) [138] could propagate disease by the transmission of their aberrant folding states, which are usually due to an increase in β-sheet structures that promote aggregation to other cellular proteins [139]. Prion-like propagation has been proposed for many of the neurodegenerative-disease-unique neurotoxic factors that are secreted extracellularly, including α-Synuclein [140], β-amyloid [141], tau [142] and TDP-43 [143]. mHTT has been likewise associated with a prion-like mode of propagation and interneuronal transmission [55,58]. In cultured cells, internalized and cytoplasmically translocated polyQ aggregates could selectively recruit the soluble cytoplasmic proteins with which they share homologous amyloidogenic sequences, with these being co-sequestered in aggresomes (which are basically aggregates of misfolded proteins) together with UPS components and cytoplasmic chaperones [122]. Structural analyses indicate that the expanded polyQ region of mHTT adopts a β-sheet structure [144]. In a *Drosophila* model of phagocytic glia clearance of engulfed neuronal mHTT aggregates, these appeared to effect a prion-like conversion of soluble, wild-type HTT in the glial cytoplasm in a manner that is dependent on the glial scavenger receptor, Draper [58]. Both synthetic polyQ oligomers as well as CSF from HD transgenic rats and human HD subjects were shown to seed mHTT aggregation in intact cells and lysates. The synthetic seeds were shown by light and ultrastructural analysis to nucleate and enhance mHTT aggregation, which is reflective of a prion-like propagation mechanism [54]. Of course, like the prion protein [119,145], mHTT could be transferable intercellularly in experimental models through both exosomes and TNTs. However, although mHTT aggregates could potentiate further aggregation and wild-type HTT could be included in these aggregates, there is no clear evidence that mHTT or its aggregates are able to impart a conformation change to wild-type HTT in a classic, prion protein-specific manner. Of course, there is also no evidence that HD could be transmissible in a prion disease-like manner between individuals. 

Despite the suggestive evidence above, whether the spread of mHTT from some initiating neurons to others plays a significant role in HD pathology remains questionable. The prion disease family member, Creutzfeldt–Jakob disease (vCJD) in humans is usually fatal within a short timespan upon onset of neurological symptoms (although others, like Kuru, have a much longer period of incubation). On the other hand, HD, particularly the adult onset form, has a typically long disease survival timespan of 10–20 years upon symptom onset. The transmission of human mHTT and the HD disease from human tissues to experimental animals has been demonstrated [55], but human-to-human transmissions via transplantation have not yet been known to occur. The notion of a prion-like propagation of tau has recently been critically appraised and questioned [146] and the notion of mHTT transmission in a prion-like manner would likewise need to be appraised. 

## 6. Epilogue

**Figure 1:**
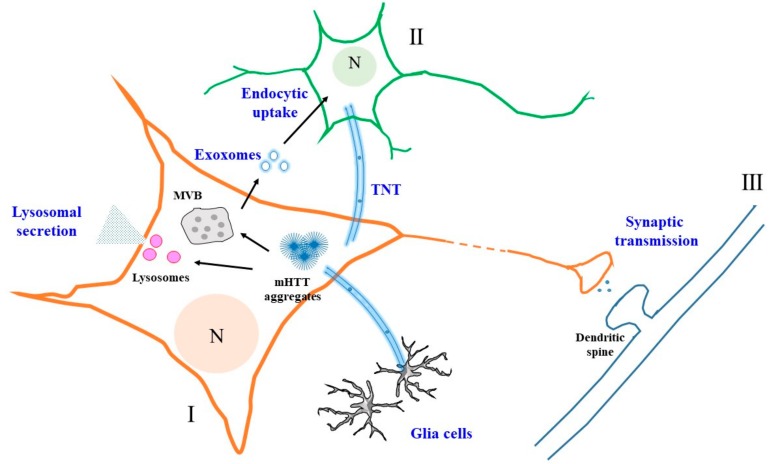
A schematic diagram illustrating the various modes of mutant Huntingtin (mHTT) unconventional secretion and intercellular propagation. Secretion from one neuron (I) could occur via exosomes when luminal vesicles from the multivesicular body (MVB) fuse with the plasma membrane. These exosomes could be taken up endocytically by another neuron (II). Secretion could also occur via a lysosome-based mechanism, with the release of non-vesicular mHTT. Interneuronal transfer, particularly between neurons (I and III) connected within a neural circuit, could occur via vesicles generated at the synaptic terminals. Intercellular transfer of mHTT aggregates could also occur via tunneling nanotubes (TNTs). N—nucleus. See text for more details.

In the paragraphs above, I outlined and discussed the current knowledge with regards to the unconventional secretion and intercellular transfer of mHTT. As for several other pathological factors of other prominent neurodegenerative disorders, cytoplasmic mHTT and its aggregates could be exported via exosomes (or other yet-unconfirmed forms of EVs), lysosome-based exocytosis and could also be transported intercellularly via TNTs. These possibilities are summarized in Figure 1. Importantly, emerging evidence suggests that mHTT aggregates, originating in a few neurons, perhaps with functional impairments, could potentially be propagated within the CNS via such intercellular transfer mechanisms. In the case of mHTT aggregates seeding the misfolding or aggregation of other mHTT oligomers or even wild-type HTT in a prion-like fashion, pathological transmission could be amplified. The latter notion would require further investigations to establish its true significance in the human disease etiology. Such investigations may benefit from the development of HD models with mutant *HTT* knock-in in large animals [147]. From another perspective, investigations could also benefit from the development of human brain organoids, particularly those that might eventually recapitulate some form of corticostriatal connections. Mosaic organoids developed from a mixture of normal and HD patient-derived induced pluripotent stem cells harboring mHTT could potentially allow a better gauge of the contribution of intercellular transfer and propagation of mHTT aggregates from diseased neurons to healthy neighbors (harboring either wild-type HTT or mHTT) and of HD pathology.

How would our accumulated knowledge on the unconventional secretion of mHTT and intercellular propagation help with therapeutic development for HD and other neurodegenerative diseases? If mHTT intercellular transport and seeding of neighboring neurons leads to mHTT accumulation that overwhelms the cellular clearance machineries, perhaps preventing such unconventional secretions could be beneficial. In this regard, we have seen how the NS and PI3K inhibitors could effectively reduce LE/Lys targeting and lysosome-based exocytosis. These and perhaps other compounds could be developed as alternatives, or as complements, to compounds that dissolve or clear mHTT aggregates. These compounds could be effectively tested in mouse HD models for possible protective effects in terms of behavioral readouts, MSN demise, as well as survival end points. In their investigations, Trajkovic et al. concluded that lysosomal secretion is not the main clearance mechanism of mHTTs [56], but lysosomes constitute a compartment in aging cells where aggregates like those of mHTT could accumulate [148]. Enhancing lysosomal function and clearance has been shown to restore the youthfulness of neural stem cells and enhance the latter’s ability to be activated. Promoting and enhancing lysosomal function could conceivably also prolong the survival of non-dividing neurons.

## Figures and Tables

**Table 1:** Major neurotoxic agents that are known to be unconventionally secreted in neurodegenerative diseases. EV—extracellular vesicle; TNT—tunneling nanotubes.

Neurodegenerative Diseases	Unconventionally Secreted Neurotoxic Agent	Known Secretion/Intercellular Transfer Mechanisms	References
Alzheimer’s disease	Aβ peptides	Exosomes	[98]
	Tau	EVs (including exosomes)	[100,101]
		TNT	[50,105]
Parkinson’s disease	α-Synuclein	Exosomes	[84,85]
		TNT	[79,97]
Amyotrophic lateral sclerosis	SOD1	Exosomes	[113]
	TDP-43	Exosomes	[114]
		TNT	[117]
	FUS	Exosomes	[115]
Huntington’s disease	mHTT	EVs (including exosomes)	[55,121]
		TNT	[57]
Prion disease	PrPSc	Exosomes	[117]
		TNT	[118]

## References

[1] Bates G.P., Dorsey R., Gusella J.F., Hayden M.R., Kay C., Leavitt B.R., Nance M., Ross C.A., Scahill R.I., Wetzel R. (2015). Huntington disease. Nat. Rev. Dis. Primers.

[2] McColgan P., Tabrizi S.J. (2018). Huntington’s disease: A clinical review. Eur. J. Neurol..

[3] Den Dunnen W.F.A. (2017). Trinucleotide repeat disorders. Handb. Clin. Neurol..

[4] Zeitlin S., Liu J.P., Chapman D.L., Papaioannou V.E., Efstratiadis A. (1995). Increased apoptosis and early embryonic lethality in mice nullizygous for the Huntington’s disease gene homologue. Nat. Genet..

[5] Nasir J., Floresco S.B., O’Kusky J.R., Diewert V.M., Richman J.M., Zeisler J., Borowski A., Marth J.D., Phillips A.G., Hayden M.R. (1995). Targeted disruption of the Huntington’s disease gene results in embryonic lethality and behavioral and morphological changes in heterozygotes. Cell.

[6] Duyao M.P., Auerbach A.B., Ryan A., Persichetti F., Barnes G.T., McNeil S.M., Ge P., Vonsattel J.P., Gusella J.F., Joyner A.L. (1995). Inactivation of the mouse Huntington’s disease gene homolog Hdh. Science.

[7] Andrade M.A., Bork P. (1995). HEAT repeats in the Huntington’s disease protein. Nat. Genet..

[8] Seong I.S., Woda J.M., Song J.J., Lloret A., Abeyrathne P.D., Woo C.J., Gregory G., Lee J.M., Wheeler V.C., Walz T. (2010). Huntingtin facilitates polycomb repressive complex 2. Hum. Mol. Genet..

[9] Ochaba J., Lukacsovich T., Csikos G., Zheng S., Margulis J., Salazar L., Mao K., Lau A.L., Yeung S.Y., Humbert S. (2014). Potential function for the Huntingtin protein as a scaffold for selective autophagy. Proc. Natl. Acad. Sci. USA..

[10] Rui Y.N., Xu Z., Patel B., Chen Z., Chen D., Tito A., David G., Sun Y., Stimming E.F., Bellen H.J. (2015). Huntingtin functions as a scaffold for selective macroautophagy. Nat. Cell Biol..

[11] Saudou F., Humbert S. (2016). The biology of Huntingtin. Neuron.

[12] Wu L.L.Y., Zhou X.F. (2009). Huntingtin associated protein 1 and its functions. Cell Adhes. Migr..

[13] Mackenzie K.D., Lim Y., Duffield M.D., Chataway T., Zhou X.F., Keating D.J. (2017). Huntingtin-associated protein-1 (HAP1) regulates endocytosis and interacts with multiple trafficking-related proteins. Cell Signal..

[14] Bhattacharyya N.P., Banerjee M., Majumder P. (2008). Huntington’s disease: Roles of huntingtin-interacting protein 1 (HIP-1) and its molecular partner HIPPI in the regulation of apoptosis and transcription. FEBS J..

[15] McGuire J.R., Rong J., Li S.H., Li X.J. (2006). Interaction of Huntingtin-associated protein-1 with kinesin light chain: Implications in intracellular trafficking in neurons. J. Biol. Chem..

[16] Caviston J.P., Ross J.L., Antony S.M., Tokito M., Holzbaur E.L.F. (2007). Huntingtin facilitates dynein/dynactin-mediated vesicle transport. Proc. Natl. Acad. Sci. USA.

[17] Steffan J.S., Kazantsev A., Spasic-Boskovic O., Greenwald M., Zhu Y.Z., Gohler H., Wanker E.E., Bates G.P., Housman D.E., Thompson L.M. (2000). The Huntington’s disease protein interacts with p53 and CREB-binding protein and represses transcription. Proc. Natl. Acad. Sci. USA.

[18] Li S.H., Cheng A.L., Zhou H., Lam S., Rao M., Li H., Li X.J. (2002). Interaction of Huntington disease protein with transcriptional activator Sp1. Mol. Cell. Biol..

[19] Zuccato C., Tartari M., Crotti A., Goffredo D., Valenza M., Conti L., Cataudella T., Leavitt B.R., Hayden M.R., Timmusk T. (2003). Huntingtin interacts with REST/NRSF to modulate the transcription of NRSE-controlled neuronal genes. Nat. Genet..

[20] Godin J.D., Colombo K., Molina-Calavita M., Keryer G., Zala D., Charrin B.C., Dietrich P., Volvert M.L., Guillemot F., Dragatsis I. (2010). Huntingtin is required for mitotic spindle orientation and mammalian neurogenesis. Neuron.

[21] Keryer G., Pineda J.R., Liot G., Kim J., Dietrich P., Benstaali C., Smith K., Cordelières F.P., Spassky N., Ferrante R.J. (2011). Ciliogenesis is regulated by a huntingtin-HAP1-PCM1 pathway and is altered in Huntington disease. J. Clin. Investig..

[22] Haremaki T., Deglincerti A., Brivanlou A.H. (2015). Huntingtin is required for ciliogenesis and neurogenesis during early Xenopus development. Dev. Biol..

[23] Karam A., Tebbe L., Weber C., Messaddeq N., Morlé L., Kessler P., Wolfrum U., Trottier Y. (2015). A novel function of Huntingtin in the cilium and retinal ciliopathy in Huntington’s disease mice. Neurobiol. Dis..

[24] Wong Y.C., Holzbaur E.L.F. (2014). The regulation of autophagosome dynamics by huntingtin and HAP1 is disrupted by expression of mutant huntingtin, leading to defective cargo degradation. J. Neurosci..

[25] Tong Y., Ha T.J., Liu L., Nishimoto A., Reiner A., Goldowitz D. (2011). Spatial and temporal requirements for huntingtin (Htt) in neuronal migration and survival during brain development. J. Neurosci..

[26] Ben M’Barek K., Pla P., Orvoen S., Benstaali C., Godin J.D., Gardier A.M., Saudou F., David D.J., Humbert S. (2013). Huntingtin mediates anxiety/depression-related behaviors and hippocampal neurogenesis. J. Neurosci..

[27] Mangiarini L., Sathasivam K., Seller M., Cozens B., Harper A., Hetherington C., Lawton M., Trottier Y., Lehrach H., Davies S.W. (1996). Exon 1 of the HD gene with an expanded CAG repeat is sufficient to cause a progressive neurological phenotype in transgenic mice. Cell.

[28] Sathasivam K., Neueder A., Gipson T.A., Landles C., Benjamin A.C., Bondulich M.K., Smith D.L., Faull R.L.M., Roos R.A.C., Howland D. (2013). Aberrant splicing of HTT generates the pathogenic exon 1 protein in Huntington disease. Proc. Natl. Acad. Sci. USA.

[29] Lunkes A., Lindenberg K.S., Ben-Haïem L., Weber C., Devys D., Landwehrmeyer G.B., Mandel J.L., Trottier Y. (2002). Proteases acting on mutant huntingtin generate cleaved products that differentially build up cytoplasmic and nuclear inclusions. Mol. Cell.

[30] Kim M., Roh J.K., Yoon B.W., Kang L., Kim Y.J., Aronin N., DiFiglia M. (2003). Huntingtin is degraded to small fragments by calpain after ischemic injury. Exp. Neurol..

[31] Gafni J., Hermel E., Young J.E., Wellington C.L., Hayden M.R., Ellerby L.M. (2004). Inhibition of calpain cleavage of huntingtin reduces toxicity: Accumulation of calpain/caspase fragments in the nucleus. J. Biol. Chem..

[32] Kim Y.J., Yi Y., Sapp E., Wang Y., Cuiffo B., Kegel K.B., Qin Z.H., Aronin N., DiFiglia M. (2001). Caspase 3-cleaved N-terminal fragments of wild-type and mutant huntingtin are present in normal and Huntington’s disease brains, associate with membranes, and undergo calpain-dependent proteolysis. Proc. Natl. Acad. Sci. USA.

[33] Graham R.K., Deng Y., Slow E.J., Haigh B., Bissada N., Lu G., Pearson J., Shehadeh J., Bertram L., Murphy Z. (2006). Cleavage at the caspase-6 site is required for neuronal dysfunction and degeneration due to mutant huntingtin. Cell.

[34] Warby S.C., Doty C.N., Graham R.K., Carroll J.B., Yang Y.Z., Singaraja R.R., Overall C.M., Hayden M.R. (2008). Activated caspase-6 and caspase-6-cleaved fragments of huntingtin specifically colocalize in the nucleus. Hum. Mol. Genet..

[35] Kim Y.J., Sapp E., Cuiffo B.G., Sobin L., Yoder J., Kegel K.B., Qin Z.H., Detloff P., Aronin N., DiFiglia M. (2006). Lysosomal proteases are involved in generation of N-terminal huntingtin fragments. Neurobiol. Dis..

[36] Miller J.P., Holcomb J., Al-Ramahi I., de Haro M., Gafni J., Zhang N., Kim E., Sanhueza M., Torcassi C., Kwak S. (2010). Matrix metalloproteinases are modifiers of huntingtin proteolysis and toxicity in Huntington’s disease. Neuron.

[37] Poirier M.A., Jiang H., Ross C.A. (2005). A structure-based analysis of huntingtin mutant polyglutamine aggregation and toxicity: Evidence for a compact beta-sheet structure. Hum. Mol. Genet..

[38] Fu Y., Wu P., Pan Y., Sun X., Yang H., Difiglia M., Lu B. (2017). A toxic mutant huntingtin species is resistant to selective autophagy. Nat. Chem. Biol..

[39] Woerner A.C., Frottin F., Hornburg D., Feng L.R., Meissner F., Patra M., Tatzelt J., Mann M., Winklhofer K.F., Hartl F.U. (2016). Cytoplasmic protein aggregates interfere with nucleocytoplasmic transport of protein and RNA. Science.

[40] Gasset-Rosa F., Chillon-Marinas C., Goginashvili A., Atwal R.S., Artates J.W., Tabet R., Wheeler V.C., Bang A.G., Cleveland D.W., Lagier-Tourenne C. (2017). Polyglutamine-expanded Huntingtin exacerbates age-related disruption of nuclear integrity and nucleocytoplasmic transport. Neuron.

[41] Zuccato C., Ciammola A., Rigamonti D., Leavitt B.R., Goffredo D., Conti L., MacDonald M.E., Friedlander R.M., Silani V., Hayden M.R. (2001). Loss of huntingtin-mediated BDNF gene transcription in Huntington’s disease. Science.

[42] Gauthier L.R., Charrin B.C., Borrell-Pagès M., Dompierre J.P., Rangone H., Cordelières F.P., De Mey J., MacDonald M.E., Lessmann V., Humbert S. (2004). Huntingtin controls neurotrophic support and survival of neurons by enhancing BDNF vesicular transport along microtubules. Cell.

[43] Liot G., Zala D., Pla P., Mottet G., Piel M., Saudou F. (2013). Mutant Huntingtin alters retrograde transport of TrkB receptors in striatal dendrites. J. Neurosci..

[44] Falk J.D., Vargiu P., Foye P.E., Usui H., Perez J., Danielson P.E., Lerner D.L., Bernal J., Sutcliffe J.G. (1999). Rhes: A striatal-specific Ras homolog related to Dexras1. J. Neurosci. Res..

[45] Subramaniam S., Sixt K.M., Barrow R., Snyder S.H. (2009). Rhes, a striatal specific protein, mediates mutant-huntingtin cytotoxicity. Science.

[46] Swarnkar S., Chen Y., Pryor W.M., Shahani N., Page D.T., Subramaniam S. (2015). Ectopic expression of the striatal-enriched GTPase Rhes elicits cerebellar degeneration and an ataxia phenotype in Huntington’s disease. Neurobiol. Dis..

[47] Phatnani H., Maniatis T. (2015). Astrocytes in neurodegenerative disease. Cold Spring Harb. Perspect. Biol..

[48] Zappulli V., Friis K.P., Fitzpatrick Z., Maguire C.A., Breakefield X.O. (2016). Extracellular vesicles and intercellular communication within the nervous system. J. Clin. Investig..

[49] Ciregia F., Urbani A., Palmisano G. (2017). Extracellular vesicles in brain tumors and neurodegenerative diseases. Front. Mol. Neurosci..

[50] Abounit S., Wu J.W., Duff K., Victoria G.S., Zurzolo C. (2016). Tunneling nanotubes: A possible highway in the spreading of tau and other prion-like proteins in neurodegenerative diseases. Prion.

[51] Victoria G.S., Zurzolo C. (2017). The spread of prion-like proteins by lysosomes and tunneling nanotubes: Implications for neurodegenerative diseases. J. Cell Biol..

[52] Pecho-Vrieseling E., Rieker C., Fuchs S., Bleckmann D., Esposito M.S., Botta P., Goldstein C., Bernhard M., Galimberti I., Müller M. (2014). Transneuronal propagation of mutant huntingtin contributes to non-cell autonomous pathology in neurons. Nat. Neurosci..

[53] Babcock D.T., Ganetzky B. (2015). Transcellular spreading of huntingtin aggregates in the Drosophila brain. Proc. Natl. Acad. Sci. USA.

[54] Tan Z., Dai W., van Erp T.G.M., Overman J., Demuro A., Digman M.A., Hatami A., Albay R., Sontag E.M., Potkin K.T. (2015). Huntington’s disease cerebrospinal fluid seeds aggregation of mutant huntingtin. Mol. Psychiatry.

[55] Jeon I., Cicchetti F., Cisbani G., Lee S., Li E., Bae J., Lee N., Li L., Im W., Kim M. (2016). Human-to-mouse prion-like propagation of mutant huntingtin protein. Acta Neuropathol..

[56] Trajkovic K., Jeong H., Krainc D. (2017). Mutant huntingtin is secreted via a late endosomal/lysosomal unconventional secretory pathway. J. Neurosci..

[57] Costanzo M., Abounit S., Marzo L., Danckaert A., Chamoun Z., Roux P., Zurzolo C. (2013). Transfer of polyglutamine aggregates in neuronal cells occurs in tunneling nanotubes. J. Cell Sci..

[58] Pearce M.M.P., Spartz E.J., Hong W., Luo L., Kopito R.R. (2015). Prion-like transmission of neuronal huntingtin aggregates to phagocytic glia in the Drosophila brain. Nat. Commun..

[59] Pandey N.K., Isas J.M., Rawat A., Lee R.V., Langen J., Pandey P., Langen R. (2018). The 17-residue-long N terminus in huntingtin controls stepwise aggregation in solution and on membranes via different mechanisms. J. Biol. Chem..

[60] McKinnon C., Tabrizi S.J. (2014). The ubiquitin-proteasome system in neurodegeneration. Antioxid. Redox Signal..

[61] Metcalf D.J., García-Arencibia M., Hochfeld W.E., Rubinsztein D.C. (2012). Autophagy and misfolded proteins in neurodegeneration. Exp. Neurol..

[62] Lee M.C.S., Miller E.A., Goldberg J., Orci L., Schekman R. (2004). Bi-directional protein transport between the ER and Golgi. Annu. Rev. Cell Dev. Biol..

[63] Chua C.E.L., Lim Y.S., Lee M.G., Tang B.L. (2012). Non-classical membrane trafficking processes galore. J. Cell. Physiol..

[64] Rabouille C., Malhotra V., Nickel W. (2012). Diversity in unconventional protein secretion. J. Cell Sci..

[65] Ng F., Tang B.L. (2016). Unconventional Protein Secretion in Animal Cells. Methods Mol. Biol..

[66] Cruz-Garcia D., Malhotra V., Curwin A.J. (2018). Unconventional protein secretion triggered by nutrient starvation. Semin. Cell Dev. Biol..

[67] Colombo M., Raposo G., Théry C. (2014). Biogenesis, secretion, and intercellular interactions of exosomes and other extracellular vesicles. Annu. Rev. Cell Dev. Biol..

[68] Sedgwick A.E., D’Souza-Schorey C. (2018). The biology of extracellular microvesicles. Traffic.

[69] Rustom A., Saffrich R., Markovic I., Walther P., Gerdes H.H. (2004). Nanotubular highways for intercellular organelle transport. Science.

[70] Agnati L.F., Fuxe K. (2014). Extracellular-vesicle type of volume transmission and tunnelling-nanotube type of wiring transmission add a new dimension to brain neuro-glial networks. Philos. Trans. R. Soc. Lond. B Biol. Sci..

[71] Janas A.M., Sapoń K., Janas T., Stowell M.H.B., Janas T. (2016). Exosomes and other extracellular vesicles in neural cells and neurodegenerative diseases. Biochim. Biophys. Acta.

[72] Levy E. (2017). Exosomes in the diseased brain: First insights from in vivo studies. Front. Neurosci..

[73] Cocucci E., Meldolesi J. (2015). Ectosomes and exosomes: Shedding the confusion between extracellular vesicles. Trends Cell Biol..

[74] Wang J., Barr M.M. (2016). Ciliary Extracellular Vesicles: Txt Msg Organelles. Cell. Mol. Neurobiol..

[75] Quezada C., Torres Á., Niechi I., Uribe D., Contreras-Duarte S., Toledo F., San Martín R., Gutiérrez J., Sobrevia L. (2018). Role of extracellular vesicles in glioma progression. Mol. Aspects Med..

[76] Ahmad T., Mukherjee S., Pattnaik B., Kumar M., Singh S., Rehman R., Tiwari B.K., Jha K.A., Barhanpurkar A.P., Wani M.R. (2014). Miro1 regulates intercellular mitochondrial transport & enhances mesenchymal stem cell rescue efficacy. EMBO J..

[77] Jiang D., Gao F., Zhang Y., Wong D.S.H., Li Q., Tse H.F., Xu G., Yu Z., Lian Q. (2016). Mitochondrial transfer of mesenchymal stem cells effectively protects corneal epithelial cells from mitochondrial damage. Cell Death Dis..

[78] Naphade S., Sharma J., Gaide Chevronnay H.P., Shook M.A., Yeagy B.A., Rocca C.J., Ur S.N., Lau A.J., Courtoy P.J., Cherqui S. (2015). Lysosomal cross-correction by hematopoietic stem cell-derived macrophages via tunneling nanotubes. Stem Cells.

[79] Abounit S., Bousset L., Loria F., Zhu S., de Chaumont F., Pieri L., Olivo-Marin J.C., Melki R., Zurzolo C. (2016). Tunneling nanotubes spread fibrillar α-synuclein by intercellular trafficking of lysosomes. EMBO J..

[80] Burré J., Sharma M., Südhof T.C. (2018). Cell biology and pathophysiology of α-Synuclein. Cold Spring Harb. Perspect. Med..

[81] Yang W., Yu S. (2017). Synucleinopathies: Common features and hippocampal manifestations. Cell. Mol. Life Sci..

[82] Lee H.J., Patel S., Lee S.J. (2005). Intravesicular localization and exocytosis of alpha-synuclein and its aggregates. J. Neurosci..

[83] Jang A., Lee H.J., Suk J.E., Jung J.W., Kim K.P., Lee S.J. (2010). Non-classical exocytosis of alpha-synuclein is sensitive to folding states and promoted under stress conditions. J. Neurochem..

[84] Emmanouilidou E., Melachroinou K., Roumeliotis T., Garbis S.D., Ntzouni M., Margaritis L.H., Stefanis L., Vekrellis K. (2010). Cell-produced alpha-synuclein is secreted in a calcium-dependent manner by exosomes and impacts neuronal survival. J. Neurosci..

[85] Danzer K.M., Kranich L.R., Ruf W.P., Cagsal-Getkin O., Winslow A.R., Zhu L., Vanderburg C.R., McLean P.J. (2012). Exosomal cell-to-cell transmission of alpha synuclein oligomers. Mol. Neurodegener..

[86] Khalaf O., Fauvet B., Oueslati A., Dikiy I., Mahul-Mellier A.L., Ruggeri F.S., Mbefo M.K., Vercruysse F., Dietler G., Lee S.J. (2014). The H50Q mutation enhances α-synuclein aggregation, secretion, and toxicity. J. Biol. Chem..

[87] Paillusson S., Clairembault T., Biraud M., Neunlist M., Derkinderen P. (2013). Activity-dependent secretion of alpha-synuclein by enteric neurons. J. Neurochem..

[88] Emmanouilidou E., Minakaki G., Keramioti M.V., Xylaki M., Balafas E., Chrysanthou-Piterou M., Kloukina I., Vekrellis K. (2016). GABA transmission via ATP-dependent K+ channels regulates α-synuclein secretion in mouse striatum. Brain.

[89] Lee H.J., Cho E.D., Lee K.W., Kim J.H., Cho S.G., Lee S.J. (2013). Autophagic failure promotes the exocytosis and intercellular transfer of α-synuclein. Exp. Mol. Med..

[90] Poehler A.M., Xiang W., Spitzer P., May V.E.L., Meixner H., Rockenstein E., Chutna O., Outeiro T.F., Winkler J., Masliah E. (2014). Autophagy modulates SNCA/α-synuclein release, thereby generating a hostile microenvironment. Autophagy.

[91] Yang Y., Qin M., Bao P., Xu W., Xu J. (2017). Secretory carrier membrane protein 5 is an autophagy inhibitor that promotes the secretion of α-synuclein via exosome. PLoS ONE.

[92] Christensen D.P., Ejlerskov P., Rasmussen I., Vilhardt F. (2016). Reciprocal signals between microglia and neurons regulate α-synuclein secretion by exophagy through a neuronal cJUN-N-terminal kinase-signaling axis. J. Neuroinflamm..

[93] Zhang S., Eitan E., Wu T.Y., Mattson M.P. (2018). Intercellular transfer of pathogenic α-synuclein by extracellular vesicles is induced by the lipid peroxidation product 4-hydroxynonenal. Neurobiol. Aging.

[94] Chai Y.J., Kim D., Park J., Zhao H., Lee S.J., Chang S. (2013). The secreted oligomeric form of α-synuclein affects multiple steps of membrane trafficking. FEBS Lett..

[95] Bieri G., Gitler A.D., Brahic M. (2018). Internalization, axonal transport and release of fibrillar forms of alpha-synuclein. Neurobiol. Dis..

[96] Oueslati A., Ximerakis M., Vekrellis K. (2014). Protein transmission, seeding and degradation: Key steps for α-Synuclein prion-like propagation. Exp. Meurol..

[97] Rostami J., Holmqvist S., Lindström V., Sigvardson J., Westermark G.T., Ingelsson M., Bergström J., Roybon L., Erlandsson A. (2017). Human astrocytes transfer aggregated alpha-Synuclein via tunneling nanotubes. J. Neurosci..

[98] Rajendran L., Honsho M., Zahn T.R., Keller P., Geiger K.D., Verkade P., Simons K. (2006). Alzheimer’s disease beta-amyloid peptides are released in association with exosomes. Proc. Natl. Acad. Sci. USA.

[99] Wang Y., Mandelkow E. (2016). Tau in physiology and pathology. Nat. Rev. Neurosci..

[100] Simón D., García-García E., Gómez-Ramos A., Falcón-Pérez J.M., Díaz-Hernández M., Hernández F., Avila J. (2012). Tau overexpression results in its secretion via membrane vesicles. Neurodegener. Dis..

[101] Chai X., Dage J.L., Citron M. (2012). Constitutive secretion of tau protein by an unconventional mechanism. Neurobiol. Dis..

[102] Kanmert D., Cantlon A., Muratore C.R., Jin M., O’Malley T.T., Lee G., Young-Pearse T.L., Selkoe D.J., Walsh D.M. (2015). C-terminally truncated Forms of Tau, but not full-Length Tau or its C-terminal fragments, are released from neurons independently of cell death. J. Neurosci..

[103] Pooler A.M., Phillips E.C., Lau D.H.W., Noble W., Hanger D.P. (2013). Physiological release of endogenous tau is stimulated by neuronal activity. EMBO Rep..

[104] Wu J.W., Hussaini S.A., Bastille I.M., Rodriguez G.A., Mrejeru A., Rilett K., Sanders D.W., Cook C., Fu H., Boonen R.A.C.M. (2016). Neuronal activity enhances tau propagation and tau pathology in vivo. Nat. Neurosci..

[105] Tardivel M., Bégard S., Bousset L., Dujardin S., Coens A., Melki R., Buée L., Colin M. (2016). Tunneling nanotube (TNT)-mediated neuron-to neuron transfer of pathological Tau protein assemblies. Acta Neuropathol. Commun..

[106] Zhao J., Li L., Leissring M.A. (2009). Insulin-degrading enzyme is exported via an unconventional protein secretion pathway. Mol. Neurodegener..

[107] Son S.M., Cha M.Y., Choi H., Kang S., Choi H., Lee M.S., Park S.A., Mook-Jung I. (2016). Insulin-degrading enzyme secretion from astrocytes is mediated by an autophagy-based unconventional secretory pathway in Alzheimer disease. Autophagy.

[108] Tamboli I.Y., Barth E., Christian L., Siepmann M., Kumar S., Singh S., Tolksdorf K., Heneka M.T., Lütjohann D., Wunderlich P. (2010). Statins promote the degradation of extracellular amyloid {beta}-peptide by microglia via stimulation of exosome-associated insulin-degrading enzyme (IDE) secretion. J. Biol. Chem..

[109] Son S.M., Kang S., Choi H., Mook-Jung I. (2015). Statins induce insulin-degrading enzyme secretion from astrocytes via an autophagy-based unconventional secretory pathway. Mol. Neurodegener..

[110] Turner B.J., Atkin J.D., Farg M.A., Zang D.W., Rembach A., Lopes E.C., Patch J.D., Hill A.F., Cheema S.S. (2005). Impaired extracellular secretion of mutant superoxide dismutase 1 associates with neurotoxicity in familial amyotrophic lateral sclerosis. J. Neurosci..

[111] Gomes C., Keller S., Altevogt P., Costa J. (2007). Evidence for secretion of Cu, Zn superoxide dismutase via exosomes from a cell model of amyotrophic lateral sclerosis. Neurosci. Lett..

[112] Cruz-Garcia D., Brouwers N., Duran J.M., Mora G., Curwin A.J., Malhotra V. (2017). A diacidic motif determines unconventional secretion of wild-type and ALS-linked mutant SOD1. J. Cell Biol..

[113] Grad L.I., Yerbury J.J., Turner B.J., Guest W.C., Pokrishevsky E., O’Neill M.A., Yanai A., Silverman J.M., Zeineddine R., Corcoran L. (2014). Intercellular propagated misfolding of wild-type Cu/Zn superoxide dismutase occurs via exosome-dependent and -independent mechanisms. Proc. Natl. Acad. Sci. USA.

[114] Iguchi Y., Eid L., Parent M., Soucy G., Bareil C., Riku Y., Kawai K., Takagi S., Yoshida M., Katsuno M. (2016). Exosome secretion is a key pathway for clearance of pathological TDP-43. Brain.

[115] Kamelgarn M., Chen J., Kuang L., Arenas A., Zhai J., Zhu H., Gal J. (2016). Proteomic analysis of FUS interacting proteins provides insights into FUS function and its role in ALS. Biochim. Biophys. Acta.

[116] Feiler M.S., Strobel B., Freischmidt A., Helferich A.M., Kappel J., Brewer B.M., Li D., Thal D.R., Walther P., Ludolph A.C. (2015). TDP-43 is intercellularly transmitted across axon terminals. J. Cell Biol..

[117] Ding X., Ma M., Teng J., Teng R.K.F., Zhou S., Yin J., Fonkem E., Huang J.H., Wu E., Wang X. (2015). Exposure to ALS-FTD-CSF generates TDP-43 aggregates in glioblastoma cells through exosomes and TNTs-like structure. Oncotarget.

[118] Fevrier B., Vilette D., Archer F., Loew D., Faigle W., Vidal M., Laude H., Raposo G. (2004). Cells release prions in association with exosomes. Proc. Natl. Acad. Sci. USA.

[119] Gousset K., Schiff E., Langevin C., Marijanovic Z., Caputo A., Browman D.T., Chenouard N., de Chaumont F., Martino A., Enninga J. (2009). Prions hijack tunnelling nanotubes for intercellular spread. Nat. Cell Biol..

[120] Vilette D., Courte J., Peyrin J.M., Coudert L., Schaeffer L., Andréoletti O., Leblanc P. (2018). Cellular mechanisms responsible for cell-to-cell spreading of prions. Cell. Mol. Life Sci..

[121] Ren P.H., Lauckner J.E., Kachirskaia I., Heuser J.E., Melki R., Kopito R.R. (2009). Cytoplasmic penetration and persistent infection of mammalian cells by polyglutamine aggregates. Nat. Cell Biol..

[122] Wild E.J., Boggio R., Langbehn D., Robertson N., Haider S., Miller J.R.C., Zetterberg H., Leavitt B.R., Kuhn R., Tabrizi S.J. (2015). Quantification of mutant huntingtin protein in cerebrospinal fluid from Huntington’s disease patients. J. Clin. Investig..

[123] Zhang X., Abels E.R., Redzic J.S., Margulis J., Finkbeiner S., Breakefield X.O. (2016). Potential transfer of polyglutamine and CAG-repeat RNA in extracellular vesicles in Huntington’s disease: Background and evaluation in cell culture. Cell. Mol. Neurobiol..

[124] Cicchetti F., Lacroix S., Cisbani G., Vallières N., Saint-Pierre M., St-Amour I., Tolouei R., Skepper J.N., Hauser R.A., Mantovani D. (2014). Mutant huntingtin is present in neuronal grafts in Huntington disease patients. Ann. Neurol..

[125] Simpson L.L. (2004). Identification of the major steps in botulinum toxin action. Annu. Rev. Pharmacol. Toxicol..

[126] Diaz-Hidalgo L., Altuntas S., Rossin F., D’Eletto M., Marsella C., Farrace M.G., Falasca L., Antonioli M., Fimia G.M., Piacentini M. (2016). Transglutaminase type 2-dependent selective recruitment of proteins into exosomes under stressful cellular conditions. Biochim. Biophys. Acta.

[127] Hong Y., Zhao T., Li X.J., Li S. (2017). Mutant huntingtin inhibits αB-crystallin expression and impairs exosome secretion from astrocytes. J. Neurosci..

[128] Claude-Taupin A., Jia J., Mudd M., Deretic V. (2017). Autophagy’s secret life: Secretion instead of degradation. Essays Biochem..

[129] Gee H.Y., Noh S.H., Tang B.L., Kim K.H., Lee M.G. (2011). Rescue of ΔF508-CFTR trafficking via a GRASP-dependent unconventional secretion pathway. Cell.

[130] Duran J.M., Anjard C., Stefan C., Loomis W.F., Malhotra V. (2010). Unconventional secretion of Acb1 is mediated by autophagosomes. J. Cell Biol..

[131] Manjithaya R., Anjard C., Loomis W.F., Subramani S. (2010). Unconventional secretion of Pichia pastoris Acb1 is dependent on GRASP protein, peroxisomal functions, and autophagosome formation. J. Cell Biol..

[132] Dupont N., Jiang S., Pilli M., Ornatowski W., Bhattacharya D., Deretic V. (2011). Autophagy-based unconventional secretory pathway for extracellular delivery of IL-1β. EMBO J..

[133] Manjithaya R., Subramani S. (2010). Role of autophagy in unconventional protein secretion. Autophagy.

[134] Heng M.Y., Duong D.K., Albin R.L., Tallaksen-Greene S.J., Hunter J.M., Lesort M.J., Osmand A., Paulson H.L., Detloff P.J. (2010). Early autophagic response in a novel knock-in model of Huntington disease. Hum. Mol. Genet..

[135] Villeneuve J., Bassaganyas L., Lepreux S., Chiritoiu M., Costet P., Ripoche J., Malhotra V., Schekman R. (2018). Unconventional secretion of FABP4 by endosomes and secretory lysosomes. J. Cell Biol..

[136] van der Sluijs P., Zibouche M., van Kerkhof P. (2013). Late steps in secretory lysosome exocytosis in cytotoxic lymphocytes. Front. Immunol..

[137] Andrews N.W. (2000). Regulated secretion of conventional lysosomes. Trends Cell Biol..

[138] Aguzzi A., Baumann F., Bremer J. (2008). The prion’s elusive reason for being. Annu. Rev. Neurosci..

[139] Prusiner S.B. (2013). Biology and genetics of prions causing neurodegeneration. Annu. Rev. Genet..

[140] Prusiner S.B., Woerman A.L., Mordes D.A., Watts J.C., Rampersaud R., Berry D.B., Patel S., Oehler A., Lowe J.K., Kravitz S.N. (2015). Evidence for α-synuclein prions causing multiple system atrophy in humans with parkinsonism. Proc. Natl. Acad. Sci. USA.

[141] Olsson T.T., Klementieva O., Gouras G.K. (2018). Prion-like seeding and nucleation of intracellular amyloid-β. Neurobiol. Dis..

[142] Ayers J.I., Giasson B.I., Borchelt D.R. (2018). Prion-like spreading in tauopathies. Biol. Psychiatry.

[143] Nonaka T., Hasegawa M. (2018). TDP-43 Prions. Cold Spring Harb. Perspect. Med..

[144] Kim M. (2013). Beta conformation of polyglutamine track revealed by a crystal structure of Huntingtin N-terminal region with insertion of three histidine residues. Prion.

[145] Hartmann A., Muth C., Dabrowski O., Krasemann S., Glatzel M. (2017). Exosomes and the Prion protein: More than one truth. Front. Neurosci..

[146] Mudher A., Colin M., Dujardin S., Medina M., Dewachter I., Alavi Naini S.M., Mandelkow E.M., Mandelkow E., Buée L., Goedert M. (2017). What is the evidence that tau pathology spreads through prion-like propagation?. Acta Neuropathol. Commun..

[147] Yan S., Tu Z., Liu Z., Fan N., Yang H., Yang S., Yang W., Zhao Y., Ouyang Z., Lai C. (2018). A Huntingtin knockin pig model recapitulates features of selective neurodegeneration in Huntington’s disease. Cell.

[148] Leeman D.S., Hebestreit K., Ruetz T., Webb A.E., McKay A., Pollina E.A., Dulken B.W., Zhao X., Yeo R.W., Ho T.T. (2018). Lysosome activation clears aggregates and enhances quiescent neural stem cell activation during aging. Science.

